# Epileptic Discharges Affect the Default Mode Network – fMRI and Intracerebral EEG Evidence

**DOI:** 10.1371/journal.pone.0068038

**Published:** 2013-06-28

**Authors:** Firas Fahoum, Rina Zelmann, Louise Tyvaert, François Dubeau, Jean Gotman

**Affiliations:** 1 Montreal Neurological Institute, McGill University, Montréal, Québec, Canada; 2 Clinical Neurophysiology Department, Roger Salengro Hospital, Lille University Medical Center, Lille, France; University of British Columbia, Canada

## Abstract

Functional neuroimaging studies of epilepsy patients often show, at the time of epileptic activity, deactivation in default mode network (DMN) regions, which is hypothesized to reflect altered consciousness. We aimed to study the metabolic and electrophysiological correlates of these changes in the DMN regions. We studied six epilepsy patients that underwent scalp EEG-fMRI and later stereotaxic intracerebral EEG (SEEG) sampling regions of DMN (posterior cingulate cortex, Pre-cuneus, inferior parietal lobule, medial prefrontal cortex and dorsolateral frontal cortex) as well as non-DMN regions. SEEG recordings were subject to frequency analyses comparing sections with interictal epileptic discharges (IED) to IED-free baselines in the IED-generating region, DMN and non-DMN regions. EEG-fMRI and SEEG were obtained at rest. During IEDs, EEG-fMRI demonstrated deactivation in various DMN nodes in 5 of 6 patients, most frequently the pre-cuneus and inferior parietal lobule, and less frequently the other DMN nodes. SEEG analyses demonstrated decrease in gamma power (50–150 Hz), and increase in the power of lower frequencies (<30 Hz) at times of IEDs, in at least one DMN node in all patients. These changes were not apparent in the non-DMN regions. We demonstrate that, at the time of IEDs, DMN regions decrease their metabolic demand and undergo an EEG change consisting of decreased gamma and increased lower frequencies. These findings, specific to DMN regions, confirm in a pathological condition a direct relationship between DMN BOLD activity and EEG activity. They indicate that epileptic activity affects the DMN, and therefore may momentarily reduce the consciousness level and cognitive reserve.

## Introduction

Functional neuroimaging studies in healthy subjects have revealed a specific network of brain regions which is active at rest and reduces its activity during goal-directed behavior, the default-mode network (DMN) [1]. It is mainly comprised of the posterior cingulate cortex (PCC), pre-cuneus (P-C), medial prefrontal and lateral parietal cortices [2]. Disruption of the activity within the DMN has been associated with many conditions in which various aspects of cognitive function are reduced, including underdeveloped connectivity in normally developing children [3] as well as disorders such as Alzheimer’s disease, schizophrenia and depression [4]–[6].

The blood oxygenation level-dependent (BOLD) signal and cerebral blood flow in DMN regions, as measured by EEG-fMRI and single-photon emission computed tomography (SPECT), have been shown to be reduced during generalized and focal epileptic activity [7]–[10]. It has been suggested that these changes are linked to the reduction in consciousness often associated with epileptic activity [7], [10]–[12]. The ‘network inhibition hypothesis’ proposes that propagation of epileptic seizures disrupts normal activity in the subcortical activating system and thus leads to decreased activation in the DMN [12], [13]. Such a disruption of the activating system could theoretically reduce activity in other resting state networks but this has not been observed.

The electrophysiological basis for DMN deactivation coinciding with epileptic activity is not clearly understood. SPECT studies showed that a decrease in cerebral blood flow concurrent with temporal lobe seizures correlates with an increase in slow waves in the frontoparietal regions [11], [14]. Intracranial investigations of complex partial seizures of temporal lobe origin linked impaired consciousness to increase in delta frequency power in DMN areas [15], [16]. However, these reports were limited to EEG frequencies up to 70 Hz, whereas the BOLD signal is linked to gamma band (50–150 Hz) power of the intracranial EEG [17]–[20]. Furthermore, EEG-fMRI studies show that DMN deactivation also accompanies generalized spike-and-wave activity or focal interictal epileptiform discharges (IEDs) of temporal, frontal and posterior quadrant origins [7]–[9], [21]. Finally, some authors have reported BOLD increases rather than decreases occurring before or during the epileptic discharges in the DMN [22], [23].

Due to these apparent inconsistencies between the results of the different research modalities, and to better establish the relationship between functional neuroimaging and electrophysiological findings, we embarked on studying the relationship between IEDs and the DMN from both perspectives. For this purpose, we studied six pharmacoresistant epilepsy patients who underwent EEG-fMRI studies and later a stereotaxic intracerebral electroencephalographic (SEEG) evaluation in various DMN regions as part of their pre-surgical work-up. These regions are not often involved in epileptogenesis and they are consequently rarely explored with intracranial recordings. Since only a small fraction of epileptic patients undergo EEG-fMRI, the group who had both an EEG fMRI study and intracerebral electrodes in DMN regions is necessarily very limited. We hypothesized that, in DMN regions, there are changes in the BOLD signal and in the SEEG power in unique frequency bands, especially gamma, coinciding with epileptic discharges occurring elsewhere in the brain.

## Methods

### Subjects

We enrolled pharmacoresistant epilepsy patients who underwent pre-surgical investigations in the years 2010 and 2011 at the Montreal Neurological Hospital and Institute and Lille University Hospital, including EEG-fMRI and high sampling rate SEEG studies. EEG-fMRI was performed prior to the invasive recordings. Electrode implantation was based on the electroclinical, neuropsychological, structural and functional imaging data; results from EEG-fMRI sometimes influenced the positioning of electrodes although a deactivation in the DMN was never an argument for implantation in these regions.

The motivation for the study was to investigate the electrophysiological basis for the BOLD signal decreases often seen in EEG-fMRI and coinciding with the BOLD increase resulting from epileptic activity. For this purpose, we included patients that fulfilled the following criteria: 1) EEG-fMRI study; 2) SEEG study with high sampling rate (1000 Hz or more) allowing to record frequencies up to 250 Hz; 3) electrodes in PCC or P-C verified with imaging; and 4) seizure onset zone outside the DMN.

Ten patients fulfilled these criteria. We excluded two patients due to continuous epileptiform activity preventing marking IED-free baselines, one patient due to lack of epileptic discharges during wakefulness, and one patient due to insufficient amount of data (see data requirements below). The clinical information and the main EEG-fMRI findings of the remaining six patients are presented in Table 1. We conducted the analyses on a single subject basis because of the small number of subjects, the variability in the electrode implantations and the variability of the location of epileptic discharges.

**Table 1:** Patient clinical data and main EEG-fMRI findings**.**

Patient	Age/Sex/onset of Epilepsy	Clinical epilepsy syndrome (lesion)	Seizureonset zone	IED recorded in EEG-fMRI	BOLD activation(t-value)	BOLD deactivation
						(DMN in bold)
						(t-value)
1	41/M/6	R FLE (non lesional)	Multifocal (R SMA+R T)	1. R T	R F (4.1), R P (4.1)	Cuneus (R>L,−5)
				2. R PO	R post inf T (5.5)	L C (−4.7)
2	21/F/16	R FLE (R SMA blurring)	R SMA+Frequent L IPL IEDs	1. Generalized spike and slow wave	L T (6.3), R T (6.0), SMA(R>L, 5.7)	**P-C (L>R,-10), L IPL(**−**10),** L Frontopolar (−9.8), **PCC (L>R,** −**8.5)**
				2. Bilateral TPO	**L IPL (6.5)**, L ant T (4.9)	R Sup P (−7.6), L Sup P (−6.4), **R IPL (**−**5.2)**
3	29/F/19	R TPO (non lesional)	R T-P junction	R TP	Mid-cingulate(R>L, 7.1), R post T (4.7)	**R IPL (**−**6.7), R DLPFC (**−**5.3), P-C (R>L,** −**4.6)**
4	22/F/20	L TLE (s/p ant T resection)	L Lateral T	L T then secondary synchronization	Mid-cingulate (L>R, 10.0)	L post T (−11.1), **L IPL (**−**9.8)**, R post T (−9.4), **MPFC (L>R,** −**8.6), L DLPFC (**−**8.0), PCC/P-C (L>R,** −**7.1)**
5	47/F/31	L Perisylvian (non lesional)	L ant Insula	L TCP electrographic seizure	Mid-cingulate (L>R, 28.2),L Insula (27.5)	**L DLPFC (**−**17.9), L IPL (**−**17.6), L PCC/P-C (**−**14.3)**
6	24/F/4	L FLE (non lesional)	L SMA	Generalized polyspike and slow wave (L>R)	L F (31.0)	L post insula (−28.5), R post insula (−27.7), **P-C (L>R,** −**26.2),** Cuneus (L>R, −25.4), **R IPL (**−**24.6)**

FLE – Frontal lobe epilepsy, TLE – Temporal lobe epilepsy, SMA – Supplementary motor area, R – Right, L – Left, Ant – Anterior, Post – Posterior, Sup – Superior, Inf – Interior, T- Temporal, F- Frontal, P- Parietal, C – Central, O – Occipital, PCC – Posterior cingulate cortex, P-C – Pre-cuneus, IPL- Inferior parietal lobule, MPFC – Medial prefrontal cortex, DLPFC – Dorsolateral prefrontal cortex.

This study was approved by the research ethics committee of the Montreal Neurological Institute and Hospital and of the research ethics committee of Lille University Hospital (Comité de protection des personnes Nord-Ouest IV) and all patients signed an informed consent.

### EEG-fMRI Acquisition and Analysis

Subjects lay at rest without performing any cognitive task in the 3T scanner (Trio, Siemens, Erlangen, Germany) for 60–120 minutes. EEG was simultaneously recorded using 25 Ag/AgCl electrodes (10–20 and 10–10 International systems) and an MR-compatible amplifier (BrainAmp, Brain Products, Munich, Germany; 5 kHz sampling). EEG was cleaned from scanner and balistocardiogram artifacts [24], [25], and IEDs were marked by an electroencephalographer. The EEG showed that the subjects were either awake or in light sleep stages during the EEG-fMRI sessions. The DMN has been shown to be active in these two states of vigilance [26], [27].

We followed the method described in details elsewhere [28]. Briefly, functional images had a voxel dimension of 3.7×3.7×3.7 mm, 33 slices, 64×64 matrix, TE = 25 ms, TR = 1900 ms. Functional images were motion corrected, spatially smoothed and analyzed using software from the Montreal Neurological Institute (http://www.bic.mni.mcgill.ca/ServicesSoftware/HomePage/).

IED timing convolved with four hemodynamic response functions (HRFs) peaking at 3, 5, 7 and 9 s after the IED was used in a general linear model analysis [29]. Final t-maps were generated by taking the maximum t-value of each voxel when using different HRFs [30].

### SEEG Recording and Contacts Selection

Depth electrodes were implanted stereotactically using an image-guidance system (SNN Neuronavigation System, Mississauga, Canada) [31]. The location of electrodes was verified with either per-implantation or post-explantation scans. Electrodes, manufactured on site (contact size 1 mm^2^, intercontact distance 5 mm) or by DIXI (DIXI Medical, France, contact size 5 mm^2^, intercontact distance 3.5 mm), had 10–15 contact leads (contact 1, the most medial). EEG was sampled at 1000 Hz (patient 5) or at 2000 Hz (patients 1–4 and 6), band-pass filtered (0.3–500 Hz except for patient 5, 0.3–250 Hz) and recorded using Harmonie (Stellate, Montreal, Canada) as described earlier [32]. The recording was performed referentially with an epidural reference electrode placed over the parietal lobe of the hemisphere contralateral to the main suspected epileptic focus. Analyses were performed on bipolar montages linking adjacent contacts. Electrode contacts with prolonged artifacts were discarded.

DMN and non-DMN contacts were defined based on the anatomical distribution of the presumed DMN, and not based on task or epileptic activity-related BOLD deactivations.

All patients had at least one electrode (2–3 contacts) in the PCC or P-C; two had bilateral PCC electrodes (patients 2 and 6). Other nodes in the DMN were sampled: All but patient 3 had inferior parietal lobule (IPL) electrodes, and two patients had electrodes in medial and dorsolateral prefrontal cortices (MPFC and DLPFC). All patients had control contacts (non-DMN) that were chosen randomly from all contacts that were outside the DMN and seizure onset zone and were clearly in the cortex. A List of DMN and non-DMN contacts in all patients is shown in Table 2.

**Table 2:** Types and number of Interictal epileptic discharges (IEDs) and baselines analyzed, and significant changes in 0–30 Hz and 40–250 Hz bands in DMN and non-DMN contacts.

Patient	SEEG IEDs analyzed	Number of IEDs/baselines	DMN contacts			non-DMN contacts		
			Significant changes at the time ofstart/center/end of IED			Significant changes at the time of start/center/end of IED		
				0–30 Hz	40–250 Hz		0–30 Hz	40–250 Hz
1	Runs of R Hippocampal spikes	246/662	**R PCC**	↑<25 Hz (start,center, end)	↓80–140 Hz (start, center, end)	**R ACC**	↑<20 Hz (center)	
			**R IPL**					
2	a. Runs of R SMA spikes	191/701	**R PCC**			**R ACC**		
			**L PCC**			**R Hippo**		
			**R IPL**			**L Hippo**		
			**L IPL**					
	b. Runs of L IPL spikes	101/701	**R PCC**			**R ACC**		↑50–60 Hz(start, center)
			**L PCC**			**R Hippo**		↑60–80 Hz (start, center)
			**R IPL**			**L Hippo**		↑60–80 Hz (start, center)
			**L IPL**	↑<25 Hz (start,center)	↓80–160 Hz (start, center)			
3	Runs of R IPL spikes	146/200	**R PCC**			**R Mid Cingulate**		
			**R IPL**	↑<10 Hz (center)	↓40–160 Hz (center)			
4	Runs of L Neocortical Temporal spikes	283/1258	**L PCC**	↑<25 Hz (start,center, end)	↓60–250 Hz (start, center, end)	**L Insula**		
			**L IPL**	↑<10 Hz (start,center)	↓80–180 Hz (start, center)	**L Cuneus**		
			**L MPFC**	↑<10 Hz (start,center)	↓60–250 Hz (start, center)			
5	Short electrographic seizures starting in L insula	136/589	**L PCC**	↑15–20 Hz (center)	↓60–100 Hz (center)	**L Inferior Occipital**		
			**L P-C**	↑15–20 Hz (center)	↓60–120 Hz (center)	**L Superior Temporal**		↑ <120 Hz (center) ↓<120 Hz (end)
			**L IPL**	↑<60 Hz (center)				
			**L DLPFC**	↑15–20 Hz (center)	↓ 40–140 Hz (center)			
6	Runs of Bilateral Diffuse spikes, maximal in L SMA	137/381	**R PCC**			**L Orbito-frontal**		↑<140 Hz (start, center)
			**L PCC**	↑<10 Hz (start,center)	↓50–150 Hz (start,center)			
			**R IPL**					

PCC – Posterior cingulate cortex, P-C – Pre-cuneus, ACC – Anterior cingulate cortex, IPL – Inferior parietal lobule, SMA – Supplementary motor area, MPFC – Medial prefrontal cortex, DLPFC – Dorsolateral prefrontal cortex, Hippo – Hippocampus.

In patient 3, the pre-implantation hypothesis based on the non-invasive evaluation, was of an epileptic generator in the posterior quadrant; therefore the patient had several electrodes sampling that area. The intracranial study showed interictal discharges originating from a large irritative zone spanning the posterior quadrant (temporo-parieto-occipital region); however the seizure onset zone did not include the IPL contacts.

We selected adjacent channels that were clearly in the grey matter. For electrodes aiming the mesial structures (PCC, P-C, MPFC, Hippocampi etc …), the three most medial bipolar channels were used, whereas for the lateral structures (IPL, DLPFC, lateral temporal neocortex, etc….), the three most lateral channels were selected for analysis. In patient 5, we used the two most mesial and lateral bipolar channels, because these were the only channels in the grey matter.

The DMN is active during wakefulness. To infer the wakeful state, in three patients simultaneous video recordings of the interictal periods analyzed were available, two patients had simultaneous recordings of electro-oculogram and electromyogram, and in one patient we used spectral analysis of the EEG [33] during daytime hours.

### Marking IEDs and Baseline in SEEG

IEDs and IED-free baselines were manually marked in the awake state, at least 4 hours from a clinical seizure. If the patient had more than one IED type, we marked the most predominant IED. In patient 2, there were two types of frequent IEDs and both were analyzed separately – if the two IED types co-occurred they were not marked. We marked all IEDs in the available data provided they occurred in the awake state. All patients had few isolated IEDs, insufficient for analysis, and therefore these were not analyzed. Five of 6 patients had frequent runs of IEDs. A run of IEDs was defined as 2 or more IEDs without any interval between them. Runs had variable duration, but usually lasted up to a few seconds each. Patient 5 had frequent electrographic seizures, without clinical symptoms and signs as seen by the simultaneous Video-EEG monitoring. The electrographic seizures lasted 5 s on average. The interictal events that were analyzed were IED runs and electrographic seizures.

Baselines had similar duration to the average IED of the patient. Baselines were marked in temporal proximity to the IEDs, where clearly there was no epileptic activity. Baselines were marked within 30 s of each IED to increase the likelihood that IEDs and baselines were from the same state of vigilance. All baseline sections within this period were included, except when the maximum number of analyzable sections (IEDs plus baselines) reached the maximum imposed by the program. When IEDs were not separated by long intervals, baseline sections were fewer. We kept enough background margins before and after the IED runs - if successive IED runs fell within 3 s from each other, they were not marked.

IED segments were compared to baselines in the IED-generating contacts as well as in DMN and non-DMN contacts. In patients 2 &3, the IPL was the spiking channel and also part of the DMN. The location of electrodes, types and numbers of IEDs and baselines are presented in Table 2.

**Figure 1:**
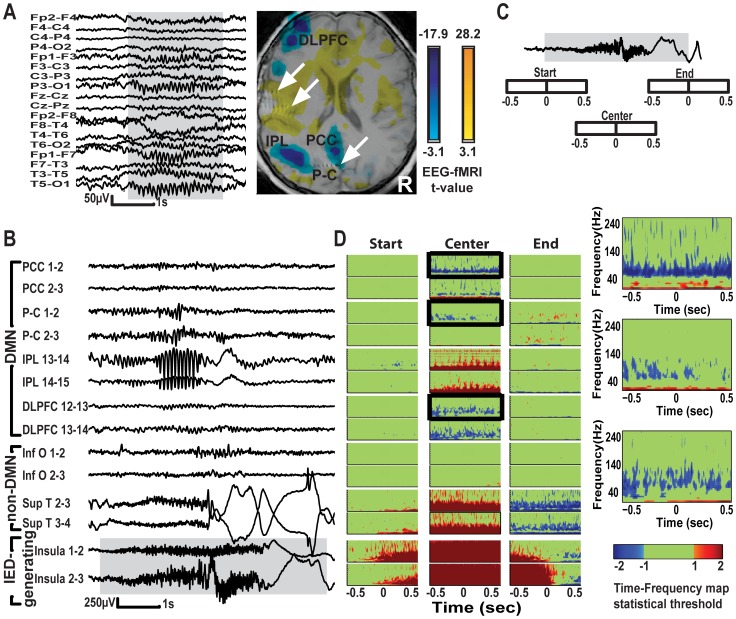
EEG-fMRI results and Time-Frequency (TF) statistical maps of SEEG recordings from patient 5. A – Scalp EEG showing electrographic seizure over perisylvian region and associated BOLD changes. Functional map is co-registered with anatomical MRI and CT showing the sampling of P-C, insula and temporal cortex by SEEG electrodes (marked by white arrows, other electrodes are not shown). Functional maps show activations in left insula and bilateral cingulate and deactivations in DMN nodes of left hemisphere. B - SEEG traces showing typical electrographic seizure originating from left insula (lower 2 traces) and SEEG in left PCC, P-C, IPL, DLPFC, inferior occipital and superior temporal regions. Each region was sampled with 2 consecutive bipolar channels 5 mm apart, and higher number denotes a more lateral channel. Note the spread of seizure activity to the IPL and temporal cortex and that the activity in IPL is identical to the activity measured on the scalp. C – Schematic representation of a typical electrographic seizure (not in scale) and 1 s epochs centered at the start, center, and end of the seizure. Epochs can overlap depending on the length of the IED. D – TF maps of statistically significant z values comparing start, center, and end of IED with baseline. They show decrease in gamma and increase in frequencies<30 Hz coinciding with the center of the seizure in PCC, P-C, DLPFC, whereas IPL and superior temporal gyrus show increase of frequencies up to 60 and 120 Hz, respectively, secondary to spread of epileptic activity. Boxed TF maps are shown in higher magnification on the right. Statistical threshold obtained after false discovery rate correction (indicated as “1” in color bar). Plots are saturated at a z value that is double the threshold value (indicated as “2”). Non-significant pixels (below false discovery rate threshold) are displayed in green.

Because IEDs had intra-subject variability in duration, the frequency analyses were performed on 1 s epochs centered at the start, center and end of the IED (see diagram in Fig. 1C). This allowed us to account for changes occurring up to 500 ms before and 500 ms after the IED.

### Spectral Analyses

Our main aim was to learn about the spectral changes, especially in the gamma band in DMN regions, which are time-locked to IEDs; therefore we used a relative time-frequency (TF) analysis. However, we also found significant changes in lower frequencies and narrower bands that are more easily demonstrated using relative power spectral density plots.

TF analysis was performed using continuous wavelet transform (WT) maps in which the complex wavelet transform was computed for each IED and baseline segment using the complex Morlet wavelet (Fb = 2, Fc1.1141; wavelet toolbox, Matlab). Wavelet power (WP) was computed as the square of the wavelet transform. Relative TF maps for each channel were obtained by dividing the median power map across IEDs by the median power map across baselines. Power (in dB) was computed as WP_dB_ = 10log_10_(WP).

Relative power spectral density computed for each IED and baseline segment using the Welch algorithm (overlap = 50%; nFFT = 64). Relative averaged power spectra were obtained by dividing the averaged spectra across IEDs by the averaged spectra across baselines.

### Statistical Comparison

Pixels of the WT maps were compared using the Mann–Whitney–Wilcoxon test. A non-parametric unpaired test was used given the lack of normality of the maps and since baselines and IEDs were uniformly selected along the EEG segments, but without one-to-one correspondence between the IEDs and baseline sections. To correct for multiple comparisons the false discovery rate procedure was applied (Matlab function fdr_bh.m) [34]. Significance level was set at 0.05.

## Results

### EEG-fMRI

**Figure 2:**
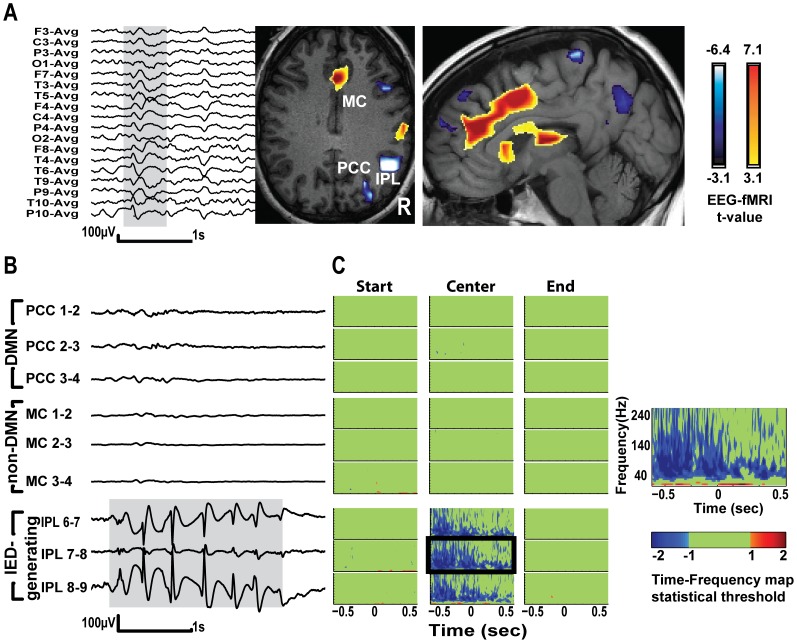
EEG-fMRI results and Time-Frequency (TF) statistical maps of SEEG recordings from patient 3. A – Scalp EEG showing right temporo-parietal IED (spike and slow wave) and the associated BOLD changes. Most significant deactivation (t = -6.7) is in right IPL and concordant with the spike field. Smaller deactivations are in posterior pre-cuneus and frontal and parietal regions ipsilateral to the IED. Activations are in the cingulate gyri, thalami and ipsilateral central region. B - SEEG traces showing typical runs of spike and slow waves in right IPL (lower 3 traces) and in right PCC and middle cingulate (MC). C – TF plots comparing IEDs and baseline in PCC, MC and the channels with IEDs (IPL). The IPL is the spiking channel and part of the DMN. TF maps centered at the center of IED run show decrease in gamma in the channels with IEDs (IPL). Boxed TF map is shown in higher magnification on the right and demonstrates power decrease in 40–160 Hz band, and increase in frequencies <30 Hz.

**Figure 3:**
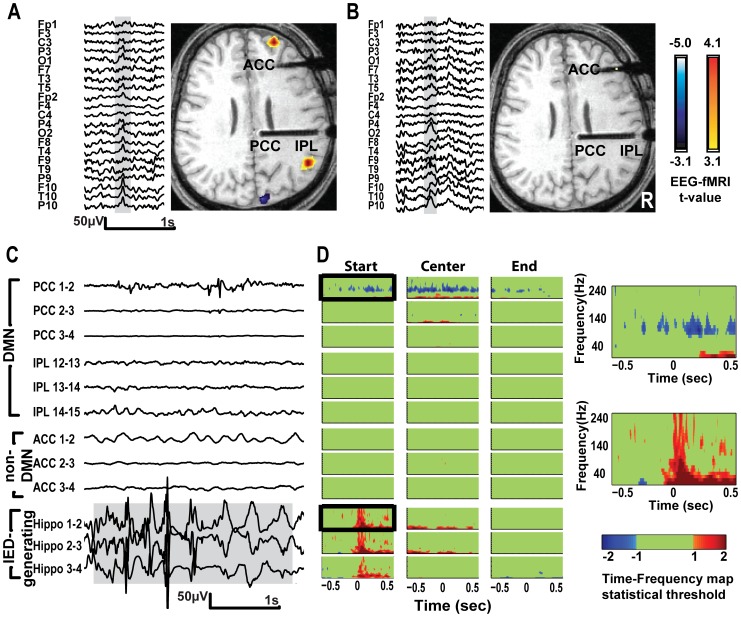
EEG-fMRI results and Time-Frequency (TF) statistical maps of SEEG recordings from patient 1. A – Scalp EEG showing right temporal IED and the associated BOLD changes. Functional map is co-registered to anatomical MRI acquired with the SEEG electrodes, showing the sampling of PCC, IPL and ACC. Functional maps show significant activations in right frontal and parietal lobes and deactivation in cuneus. B - Right posterior temporal-occipital sharp wave was associated with right inferior temporal activation and left central deactivation (not shown in figure). EEG-fMRI did not show DMN deactivation in A&B. C - SEEG traces showing typical runs of hippocampal IEDs (lower 3 traces) and SEEG in PCC, IPL and ACC. Most medial channel in PCC (upper trace) shows propagation of epileptic activity. D – TF plots comparing IEDs with baseline in PCC, IPL, ACC and the IED-generating channel (hippocampus). TF maps are centered at the start, center and end of IED, showing statistically significant increase in frequencies <30 Hz in the PCC (reflecting spike propagation) and decrease in gamma coinciding with the hippocampal IED. Boxed TF maps shown in higher magnification on the right illustrate power change in PCC and IED-generating channel. Power decrease in PCC is in 80–140 Hz band, and starts 250 ms before IED onset.

Results are presented in Table 1. All patients had scalp IED-correlated activations in the distribution of the presumed epileptic network. Five of six patients had deactivations in DMN regions; patient 1 showed no deactivation in any node of the DMN. Among the five patients with DMN deactivation, the most involved DMN nodes were the IPL and P-C (5/5 patients for both). The PCC (3/5), DLPFC (3/5) and MPFC (1/5) were deactivated less frequently. The IPL was deactivated ipsilateral to the IED in 4/5 patients and DLPFC was also deactivated ipsilaterally in 3/5. The midline nodes (PCC, P-C and MPFC) were usually involved bilaterally with higher t-value ipsilateral to the IED. Fig. 1A and Fig. 2A show examples of ipsilateral and midline DMN nodes deactivation whereas in Fig. 3A and Fig. 3B there is no significant decrease in BOLD signal in those regions.

Patient 2 had two types of IEDs in the scanner, and these were associated with different patterns of DMN nodes deactivation. Generalized IEDs were associated with more widespread and bilateral deactivation in DMN nodes, whereas bilateral posterior IEDs were associated with more restricted DMN deactivation limited to IPL unilaterally (Table 1).

### SEEG

In 4/6 patients, the SEEG implantation involved the anatomical regions of DMN nodes unilaterally (Fig. 1A & Fig. 3A–B), whereas in two patients (2 and 6) the sampling of DMN regions was bilateral. Results are summarized in Table 2, showing changes in frequencies in DMN and non-DMN locations at times of IEDs compared to baselines. Only in DMN regions, there is a decrease in gamma band power, and increase in the power of the lower frequencies (<30 Hz) at times of IEDs, in at least one DMN node in all six patients. Here are examples showing the variability of the spectral changes, and the EEG-fMRI findings, among the different patients:

Patient 5– On scalp EEG, this patient had frequent electrographic seizures over the left perisylvian region associated with BOLD increases in the left insula and mid-cingulate gyri and with deactivations following the distribution of the left hemispheric DMN (Fig. 1A). The SEEG study showed short electrographic seizures originating from the left insula (Fig. 1B). The TF representation of SEEG activity in DMN contacts at the time of IEDs compared to baseline, shows significant decrease in gamma power in PCC, P-C and DLPFC and increase in power of frequencies <30 Hz coinciding with the center of the seizure (Fig. 1D and boxed maps) whereas in the IPL there is an increase in the power up to 60 Hz in this region due to a spread of epileptic activity (Fig. 1B&D). The gamma decrease occurs in different bands within the gamma range in the different nodes: 60–100 Hz in PCC, 60–120 Hz in P-C and 40–140 Hz in DLPFC. The non-DMN regions did not show decrease in gamma: there is no significant change in the inferior occipital region while in the ipsilateral superior temporal gyrus there is propagation of the epileptic activity causing an increase in power up to 120 Hz at the center of the seizure and a long-lasting decrease at the end coinciding with high amplitude post ictal slow waves (Fig. 1B).

Patient 1 - EEG-fMRI did not show any significant BOLD changes in DMN regions (Fig. 3A–B). SEEG study disclosed multifocal epileptogenicity in the right SMA, inferior temporal cortex and hippocampus. The most prevalent IEDs were runs of hippocampal spikes and slow waves usually spreading to the PCC (Fig. 3C, three lowest traces and upper trace). The most medial channel in PCC shows decrease in 80–140 Hz band starting 250 ms before the IED starts and lasting until the IED ends, and increase in frequencies <30 Hz (Fig. 3D, and first boxed map). In the adjacent PCC contacts and IPL contacts, there was no decrease in gamma.

Patient 3– EEG-fMRI and SEEG studies disclosed an epileptic generator in the right temporo-parietal junction. Most significant BOLD deactivations were found in that region and in concordance with the IED field, whereas most significant activations were in the anterior and middle cingulate gyrus, bilaterally. A smaller cluster of deactivation was in the posterior P-C (Fig. 2A), but this area was not sampled by an SEEG electrode. TF maps show gamma decrease (50–140 Hz) and increase in lower frequencies in IPL, at the center of IED compared to baseline.

The 3 other patients (2, 4 and 6) showed significant decrease in gamma and increase in lower frequencies in at least one DMN region coinciding with IED times. Their findings are summarized in Table 2. In patient 2, significant changes are found in IPL (channel with IEDs). In patient 4, changes were found in multiple nodes of DMN (in PCC, MPFC and IPL) as well as in the IED-generating contacts in the temporal neocortex. In this patient, gamma decrease started up to 400 ms prior to IED onset. Finally, in patient 6, significant changes are found in PCC.

**Figure 4:**
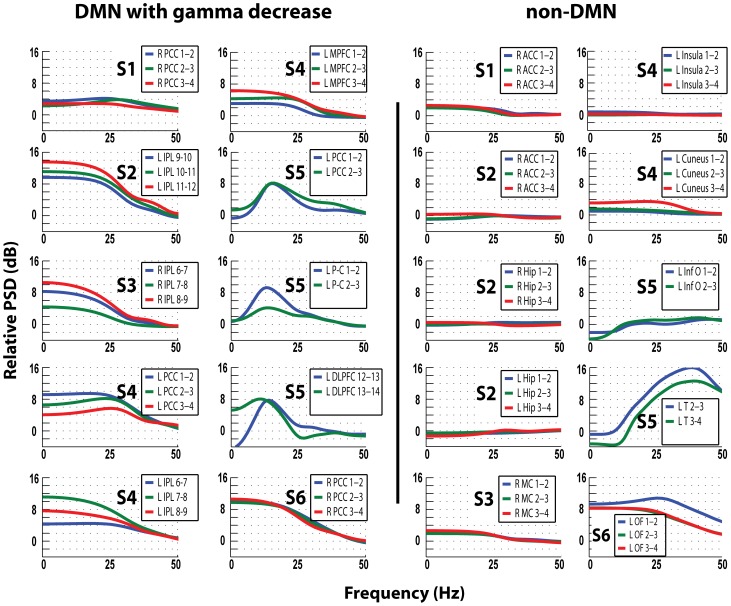
Relative power spectral density in dB, for frequencies 0–50 Hz. Power spectra in the DMN regions with gamma decrease and the non-DMN regions in all patients comparing 1 s epochs at the center of IED to baseline. Blue trace is the most medial, followed laterally by the green and red traces. S1–S6 relates to patients 1–6. Note the power increase in frequencies <30 Hz in all DMN regions with gamma decrease, but only in 2/10 non-DMN locations: Temporal cortex in patient 5 shows increase in low frequencies, secondary to propagation of epileptic activity (Fig. 1B), and LOF contacts of patient 6 also due to propagation of IEDs originating from left SMA (not shown). Comparing Fig. 4 and 5 shows an inverse relationship between the decrease in gamma and the increase in the lower frequencies in many DMN nodes: contacts that showed more gamma decrease also had more increase in lower frequencies: For examples, compare the responses in L PCC in patient 4 and L P-C in patient 5.

**Figure 5:**
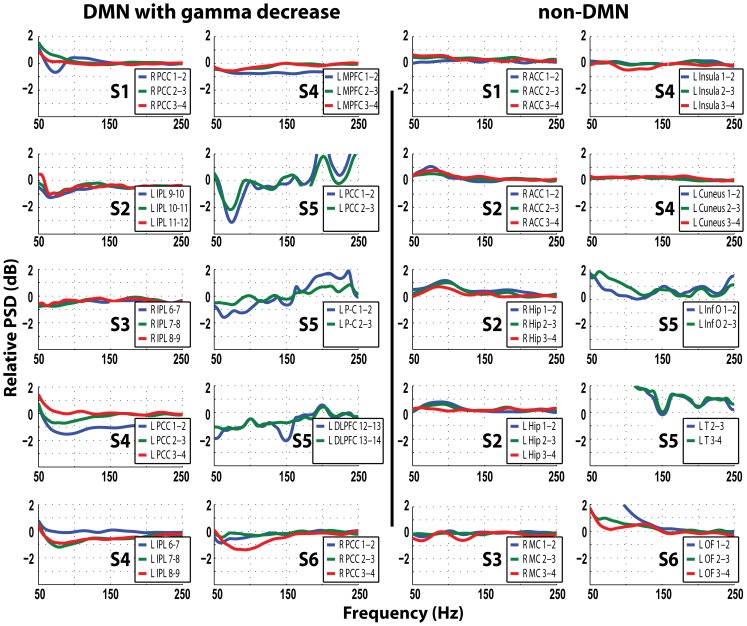
Relative power spectral density in dB, for frequencies 50–250 Hz. Power spectra in the DMN regions with gamma decrease and the non-DMN regions in all patients comparing 1 s epochs at the center of IED to baseline. Blue trace is the most medial, followed laterally by the green and red traces. S1–S6 relates to patients 1–6. Note the consistency of gamma decrease in DMN regions and the lack of similar spectral changes in non-DMN contacts.

The magnitude of the increase in power of lower frequencies was larger than the magnitude of decrease in gamma. For the clarity of the demonstration we present the data in two sets of power spectral density plots, one for lower (0–50 Hz, Fig. 4) and one for higher frequencies (50–250 Hz, Fig. 5). We found that in DMN regions, gamma decrease is accompanied by power increase in frequencies <30 Hz (Fig. 4): maximal power increase in the delta-theta range in patients 2, 3, 4 and 6, whereas in the PCC of patients 1 and 4 the increase peaked at 20 Hz, and in patient 5 all DMN nodes with gamma decrease had power increase in the beta range (15–20 Hz). In 8/10 DMN nodes with gamma decrease there is an inverse relationship between these two bandwidths – the contacts showing higher power increase in lower frequencies had stronger gamma decrease (for examples compare Fig. 4 & Fig. 5 for the responses in L PCC in patient 4 and L P-C in patient 5).

## Discussion

Impaired consciousness is a key phenomenon during epileptic seizures, and it is usually explained by the extension and intensity of the on-going epileptic activity. Using EEG- fMRI and SPECT some authors proposed that the alteration in consciousness corresponds to a decrease in the metabolism and blood flow in DMN regions [7], [8], [11]. During generalized or complex partial seizures, the ongoing ictal epileptic activity spreads to the diencephalic reticular formation [12], [13], [35] and this eventually causes augmentation of slow waves in DMN regions [15], [16].

However, and despite the advances in understanding of these brain mechanisms, several issues remain unresolved: 1. Similar deactivations accompany the brief interictal epileptic activity as well [7]–[9], [21] 2. DMN deactivation also accompanies focal IEDs originating from extra-temporal regions [21] 3. Despite the fact that virtually all work in humans relating BOLD and electrophysiology was performed on invasive recordings from epileptic patients, while performing cognitive tasks and excluding epochs and regions involved with epileptic discharges, very few studies have been done on the unique relation between epileptic discharges and DMN regions and possible relation to associated behavioral changes of reduced consciousness [4]. The available data in this matter are limited to conventional EEG frequencies (<70 Hz) [15], [16], discarding the higher frequencies that are relevant to the known coupling with the BOLD signal.

Our work, using two modalities of scalp EEG-correlated fMRI and SEEG, tried to address this issue from two angles while relying on the unique strengths of each method. EEG-fMRI has the advantage of scanning the whole brain simultaneously with excellent spatial resolution, whereas the SEEG has very good temporal resolution and can identify very small and complex changes in electrophysiological properties that may have metabolic correlates not visible with fMRI. The main limitations of our study are the small patient sample, and the under-sampling of the brain by the SEEG technique. The small patient group originates from the generally low epileptogenicity of regions of the DMN and therefore their infrequent implantation, and from the requirement that patients had an EEG-fMRI study as well as such an implantation.

With EEG-fMRI we saw that focal IEDs originating from different and distant brain regions coincide with a decrease in BOLD signal in various nodes of the DMN, in agreement with previous reports. We found that the involvement of the different nodes is unique for each patient, and it tends to occur more ipsilateral to the epileptic focus. Recent EEG-fMRI group analysis study suggested that IEDs originating from different brain regions deactivated the DMN in a syndrome-specific way [21]. Our sample is too small to support such a notion but we did find BOLD changes in most of our patients. We found that the P-C and IPL are the nodes most commonly showing BOLD decreases at the time of epileptic activity whereas the PCC, DLPFC and MPFC are also deactivated but less frequently.

Using frequency analysis of SEEG data from several DMN regions, we compared IED epochs to temporally adjacent IED-free baselines and we were able to capture a relatively stereotypical electrophysiological signature in at least one DMN region in all patients. In general, at the time of IEDs, there was a power decrease in gamma and increase in frequencies lower than 30 Hz. Non-DMN regions did not show similar phenomena at the time of IEDs. The changes in the DMN were at the time of epileptic activity while subjects were ‘at rest’ and not engaged in any cognitive task. This differs from reports of in which gamma was reduced at the time of cognitive tasks, in which epochs of epileptic activity were specifically excluded [36]–[38].

One patient did not have IED-related BOLD decreases in any DMN node. Relying only on BOLD measurements is yet limited because the BOLD signal is indirectly related to neuronal activity, and it represents the total activity in a certain region [17]. In that patient, the SEEG study demonstrated very focal frequency changes in the PCC, in a very similar pattern to what we have seen in the rest of our patient sample. This example highlights the paramount contribution of direct electrophysiological recordings, as we have used in our study. We will now discuss the different aspects of these findings.

### Relation to Type of IED

We analyzed runs of IEDs in five patients and short electrographic seizures in one patient. None had a sufficient number of isolated spikes for analysis, therefore our conclusions are limited to runs of interictal epileptic discharges, and might not be generalizable to isolated IEDs. The fact that all our patients had runs of IEDs could be explained by the bias in analyzing the patients that require intracranial work-up. These patients have long standing and severe drug-resistant epilepsy that is usually accompanied by highly active epileptic activity.

In 4 of 6 cases the IEDs originated from the seizure onset zone, one patient had IEDs originating from irritative tissue distant from the seizure onset zone, and one patient had two types of IEDs analyzed: one from the seizure onset zone and another from outside this region. Interestingly, in the latter patient, the decrease in gamma accompanied only IEDs located outside the seizure onset zone. These results suggest that epileptic activity with variable morphology and originating from different brain regions, and not limited to the seizure onset zone, is associated with significant changes in specific frequencies in DMN regions.

### Effect on Different DMN Nodes

Based on our inclusion criteria, all patients had at least one electrode sampling the PCC or P-C, which are considered one functional unit and a major hub in the DMN [2], [39]. The PCC/P-C and IPL, which were the nodes most commonly sampled (each area sampled 9 times in the six patients), showed IED-correlated gamma decrease in 5/9 and 3/9 cases respectively. Changes were most evident in the most medial contacts of PCC/P-C and the lateral contacts of the IPL and usually involved two or more adjacent channels. In 3 cases, the significant changes were very clear in a single channel and minimally or not evident in the adjacent channel 5 mm away. This later observation could be due to changes that are very focal in nature or due to the imprecision in the localization of the intracerebral electrodes.

The IPL showed gamma decrease only when the IEDs originated from a nearby region (IED significantly involving the same electrode; patients 2&3), whereas the PCC, P-C, and the frontal nodes had decreased gamma also with distant IEDs. The spectral changes in the channel with IED (IPL) in patients 2&3 could be interpreted as secondary to the slow wave component of the spike and wave discharge, because the slow wave could suppress high frequencies [40]. However we did not find these changes in other electrodes that had discharges with slow waves (for example patient 1, Fig. 3C–D); moreover, these changes were not found in the analyses of the start and end of the IED, despite the fact that these epochs also included slow waves. Finally, the SEEG exploration showed that the IEDs that were captured by the IPL electrodes were part of a larger epileptogenic tissue, therefore one cannot exclude that the IEDs captured by these electrodes propagated from nearby regions. These points argue that the spectral changes captured in the IPL electrodes were not different from the changes documented in other DMN nodes.

No decrease in gamma was seen in non-DMN regions including dorsal anterior and middle cingulate, inferior occipital, orbitofrontal, cuneus and insula. In patient 5, the lateral temporal cortex showed gamma decrease coinciding with high amplitude post-ictal slow waves, whereas in patient 4, the lateral temporal cortex, along with different DMN nodes, showed decreased gamma throughout the time of the IED runs. The lateral temporal cortex has been shown to be deactivated in EEG-fMRI studies of generalized and focal epilepsies [7], [21], and is considered by many authors as part of DMN [38], [41].

### Temporal Dynamics of IED-correlated Decrease in Gamma

In patients 2, 5 and 6 the decrease in gamma began with the IED start until the center of the IED and subsided before the end of IED. In patients 3 it occurred only at the center of the epileptic activity. In patients 1 and 4 the gamma decrease occurred slightly prior to the IED start. These results should be interpreted with caution, because it might be concluded that the decrease in gamma in DMN nodes precedes the epileptic activity, but it is possible that a low amplitude epileptic activity started prior to our manual marking, or alternatively another IED in a non-sampled region took place before our marked IED. These options cannot be entirely excluded. Ossandon and colleagues have demonstrated that with visual search task, the time course of gamma decrease differed across different DMN nodes – occurring earlier in IPL than in PCC and MPFC [38]. In our patients the coverage of different DMN nodes varied from one patient to another, and therefore it is hard to conclude regarding a temporal hierarchy of involvement within the different DMN nodes in association with epileptic activity.

### Decrease in Gamma is Not Uniform in Various Nodes

The frequencies within the gamma band that showed significant decreases were different across subjects as well as across different DMN nodes within the same subject. This is in agreement with the cognitive neuroscience literature showing various decreases in different sub-frequencies within the gamma range. Recent work systematically examining this point provides evidence that changes in gamma are non-uniform and depend on the task and anatomical location of the node [42].

It is suggested that different gamma sub-bands emerge from different mechanisms, as oscillations in the 30–90 Hz range show coupling with the theta rhythm, whereas no such relationship is found with faster oscillations (90–150 Hz) in the rat hippocampus [43]. The fast gamma power (>80 Hz), however, shows tight coupling with the neuronal spiking rates in the visual cortex of the monkey, whereas the slower gamma does not [44].

### Increase in Power of Lower Frequencies

We have observed increases in frequencies lower than 30 Hz in all DMN regions with decrease in gamma. Furthermore, in most cases, the magnitude of gamma decrease was inversely related to the power increase of lower frequencies. This reciprocal relationship, especially between gamma and theta and alpha, was demonstrated in some DMN regions while performing various cognitive tasks [36], [38], [45], [46]. In the case of epileptic activity, another explanation could still be suggested: direct spread of IEDs could account for increase in power in various bandwidths including lower frequencies, as we have seen in several regions. In one patient the IED clearly propagated from the hippocampus to the PCC (Example in Fig. 3C and average of all IEDs in time frequency maps in Fig. 3D), in keeping with the known anatomical connectivity between these two areas [47]. The lack of evidence for direct propagation of epileptic activity in all other DMN nodes with decreased gamma is in keeping with the notion that increase in power of lower frequencies is part of a complex phenomenon in the DMN at the time of IEDs. Such changes, including decreased gamma power and an increase in lower frequencies, are possibly mediated by propagation of epileptic activity to the diencephalic reticular formation as previously hypothesized [12], [13].

### Conclusions

Runs of IEDs and electrographic seizures originating from various brain regions, while they are not associated with overt clinical manifestations, indirectly affect DMN nodes by decreasing the gamma and increasing the power in lower frequencies, thus reducing the metabolic demands in these regions and possibly reducing the level of consciousness. These findings shed light on a possible mechanism by which focal interictal epileptic activity may momentarily reduce attention and cognitive reserve and may have a more substantial effect on brain function than previously thought. The findings also strengthen the relationship between DMN deactivation, decreased gamma, and increased lower frequencies in the EEG.
